# Attentional shifting as a potential explanatory variable in aphasic anomia

**DOI:** 10.3389/fnhum.2026.1833957

**Published:** 2026-07-17

**Authors:** Christina Sen, JoAnn P. Silkes

**Affiliations:** 1 Joint Doctoral Program in Language and Communicative Disorders, San Diego State University and University of California San Diego, San Diego, CA, United States; 2 School of Speech, Language, and Hearing Sciences, San Diego State University, San Diego, CA, United States

**Keywords:** anomia, aphasia, attention, attentional blink, cognition

## Abstract

**Introduction:**

In addition to the language impairments experienced by people with aphasia (PWA), such as lexical access deficits, aphasia is often also accompanied by impaired attention, working memory, and verbal short-term memory. Growing evidence also suggests that difficulties in lexical access may be related to impaired domain-general cognitive processes, such as attention switching and processing speed.

**Methods:**

This study explored attentional shifting and processing speed in people with aphasia (*n* = 14) and age-matched controls (*n* = 18) using an Attentional Blink paradigm with both linguistic stimuli (letters) and nonlinguistic stimuli (shapes), along with tasks assessing overall processing speed.

**Results:**

Compared to controls, PWA showed impaired attentional blink performance in the linguistic condition (*F*_1,20_ = 7.35, *p* = 0.01) and also impaired processing speed [*χ*^2^ (1) = 13.38, *p* < 0.05]. In addition, an exploratory mediation analysis revealed a potential association between severity of lexical access impairments and performance on the attentional blink task, when accounting for verbal short-term memory abilities. The nonlinguistic condition was found to be too difficult for the control participants to address the experimental questions, but identified issues to be addressed in future studies with this focus.

**Conclusion:**

These findings suggest that attentional shifting may play a foundational role in aphasic language processing and impairment. Further investigation is needed to understand the role of processing speed and whether attention shifting impairments are language-specific or extend to non-linguistic attentional shifting, as well.

## 1 Introduction

Aphasia is historically known as a language disorder, resulting in heterogenous impairments in language processes such as word retrieval (i.e., lexical access), auditory comprehension, reading, and writing (Murray, 2002). Contemporary research, however, has demonstrated that this definition of aphasia is limited in that it does not account for the accumulating evidence that broader cognitive processes, not just language, are also impacted in people with aphasia (e.g., Villard and Kiran, 2017). For instance, numerous studies have shown that people with aphasia (PWA) also demonstrate impairments on tasks specifically tapping attention (Murray, 2012; Villard and Kiran, 2017), working memory (Kim et al., 2018; C. D. Martin et al., 2018; Minkina et al., 2017, 2018), executive function/cognitive control (Kuzmina and Weekes, 2017) and processing speed (Faroqi-Shah and Gehman, 2021; Yoo et al., 2022; for a full review, see Varkanitsa et al., 2023). These findings have led researchers to propose that language impairments found in aphasia originate, at least in part, from deficits in foundational cognitive mechanisms, specifically in attentional processes (Hula and McNeil, 2008; McNeil et al., 1991; Silkes et al., 2004; Tseng et al., 1993).

While there are many forms of attention (e.g., selective attention, sustained attention; Connor and Fucetola, 2011), one of the pertinent aspects of attention for language processes is rapid *attention shifting.* Attention shifting (also known as alternating or switching attention) refers to the process of shifting attention from one goal, task, or stimulus to another, and can be considered a form of cognitive control used to volitionally control and coordinate processing (Aljahlan and Spaulding, 2019; Hall and Johnson, 2004; Miyake et al., 2000; Posner and Petersen, 1990). When listening to sentences, for example, the listener must shift attention rapidly as the incoming linguistic elements (e.g., lexical-semantics, syntax) unfold (Taube-Schiff and Segalowitz, 2005). In terms of language output, the speaker must shift attention as they quickly select appropriate, relevant words and incorporate them into syntactic structures in the context of moving rapidly through constructing a message (Laganaro et al., 2019). In studies using linguistic and nonlinguistic tasks, some PWA have shown impaired attention shifting abilities compared to control participants (Chiou and Kennedy, 2009; Helm-Estabrooks, 2002; Kuptsova et al., 2023; Peristeri et al., 2020). For instance, Chiou and Kennedy (2009) explored attention shifting skills in PWA and neurotypical controls using a Go/No-go task with letter stimuli (Drewe, 1975). The PWA demonstrated slower attention shifting abilities and greater response errors than controls. This pattern of slowed attention shifting in aphasia was also demonstrated by Kuptsova et al. (2023) using a nonverbal (i.e., nonlinguistic) auditory attention shifting task.

As is clear from these studies (Chiou and Kennedy, 2009; Kuptsova et al., 2023), the debate about the role of attention in aphasia extends beyond just its influence on language abilities. Attention is not language-specific; it is foundational to all forms of cognitive processing, leading some to investigate whether the attention impairments seen in aphasia relate only to language processing or are domain-general, extending across modalities. The evidence on this topic is inconsistent, with some studies finding impairments in various aspects of nonlinguistic attention in aphasia (e.g., Hula et al., 2007; King and Hux, 1996; Murray, 2000; Murray et al., 1997; Lee et al., 2020; Yao and Zhou, 2023) and others concluding that nonlinguistic attention remains unchanged (e.g., Hibshman and Riley, 2024; Laures et al., 2003). An additional layer of complexity is that performance on linguistic and nonlinguistic tasks may differ between monolingual and bilingual people with aphasia (Dash and Kar, 2014; Gray, 2020; Gray and Kiran, 2016, 2019). Although bilingualism is not the focus of the present study, it is an important factor to consider when interpreting task performance in aphasia and is increasingly being examined in this context (e.g., Andrade et al., 2025; Dash et al., 2020; Nair et al., 2021).

The question of the influence of broader cognitive processing has practical implications for aphasia treatment: if domain-general cognitive impairments are present in aphasia, then there may be a role for interventions that target these broader processes that support, but are not limited to, language processing. If this is the case, these interventions may have the potential to be more efficient and/or effective in remediating aphasia or, at least, may provide new avenues to supplement linguistically-based aphasia treatments in meaningful ways.

Another aspect of general cognitive function that was implicated in aphasia by the studies discussed above (Chiou and Kennedy, 2009; Kuptsova et al., 2023) is speed of processing, with some PWA showing longer response times to complete the attention switching tasks. Other studies have also pointed to processing speed as a critical factor in the linguistic and cognitive impairments in aphasia, with PWA often demonstrating slowed response and reaction times across linguistic and nonlinguistic tasks (Faroqi-Shah and Gehman, 2021; Yoo et al., 2022). For instance, Yoo et al. (2022) found that PWA showed significantly longer response times on processing speed tasks compared to healthy control participants and to participants with left hemisphere damage without aphasia. Furthermore, the PWA did not show significant differences between their performance on the linguistic tasks compared to the nonlinguistic tasks, across a range of low-to-high cognitive complexity tasks, suggesting that slowed processing speed is domain general. Given that processing speed and attentional shifting are closely coordinated and interdependent functions (Stawski et al., 2013; Tsiakiri et al., 2024), individuals with deficits in one function may be particularly prone to deficits in the other. Therefore, analyzing processing speed in connection to attentional shifting abilities may help to uncover more about the underlying mechanisms of impairment in aphasia.

With this evidence for impairments in linguistic and nonlinguistic attentional shifting and processing speed in mind, in the present study we investigated rapid attention shifting abilities in PWA and controls as indexed by a phenomenon known as the *attentional blink* using both linguistic and nonlinguistic stimuli. Additionally, to explore the possible roles of attention shifting versus processing speed in aphasia, we used a rapid target detection task to understand how attentional shifting performance, as measured by the attentional blink, relates to a person’s overall processing speed.

The attentional blink is a behavioral phenomenon that occurs during a visual recognition task (i.e., Rapid Serial Visual Presentation, or RSVP) in which simple stimuli, such as letters, are flashed on a computer screen (Raymond et al., 1992). The participants are typically asked to simultaneously pay attention to two targets (T1 and T2) amongst distractors, and report them at the end of each trial. An attentional blink occurs when T2 is presented very soon after T1 and, as a result of the inability to shift attention quickly enough, the participant fails to detect T2 (e.g., Grassi et al., 2021; Raymond et al., 1992); however, if T1 and T2 are further apart in the sequence of stimuli, then participants are more likely to report seeing T2. This recovery of T2 perception after a delay reflects a transient attention cost that has resolved over time (MacLean and Arnell, 2012), a phenomenon that has been replicated in many experiments since its initial observation (e.g., Di Lollo et al., 2005; Martens and Wyble, 2010; Shapiro et al., 1997). The attentional blink phenomenon is thought to reveal a human processing limitation in allocating attentional resources (e.g., Broadbent, 1958; Chun and Potter, 1995; Dehaene et al., 2003; Isaak et al., 1999; Jolicœur and Dell’Acqua, 1999; Miyano, 2021) and/or temporal constraints in task shifting (Di Lollo et al., 2005; Kawahara et al., 2003). Regardless of the various theoretical accounts (for more discussion, see Martens and Wyble, 2010), the attentional blink paradigm can be used as a tool to understand rapid attention shifting abilities. Unlike other available measures of attention shifting (e.g., the Wisconsin Card Sorting Test, Grant and Berg, 1993; Trail Making Test, Bowie and Harvey, 2006), it does so in a manner that engages precise manipulation of temporal sensitivity while minimizing additional executive function requirements.

While the attentional blink phenomenon and its subsequent theories of processing were originally explored in neurotypical populations (e.g., Raymond et al., 1992; Ward et al., 1996), they have also been tested in populations with neurological disorders. For example, Rizzo et al. (2001) examined the attentional blink in a sample of people with unilateral brain lesions over a wide range of locations in both hemispheres and compared them with controls without brain lesions. Participants with brain lesions showed an increased attentional blink effect compared to the control group, leading these authors to conclude that focal lesions result in impaired attention shifting. In populations with developmental disorders, children and adults with dyslexia have been shown to have significantly different attentional blink effects compared to peers without dyslexia (Hari et al., 1999; Lacroix et al., 2005; Visser et al., 2004). Hari et al. (1999) found adults with dyslexia to have longer attentional blink effects, noted as “attentional dwell time,” which they suggested reflects a deficit in the timing of re-engaging attention to rapidly presented stimuli. Lum et al. (2007) explored the attentional blink in people with Specific Language Impairment (SLI; now known as Developmental Language Disorder) and found that they also demonstrated longer attentional blinks compared to neurotypical control participants, positing that people with SLI also have difficulty with rapidly engaging and disengaging attention. Taken together, these findings in different populations highlight that attentional shifting is commonly impaired in the presence of language-related disorders. As deficits in processing speed and delayed response times have also been found in aphasia (i.e., Faroqi-Shah and Gehman, 2021; Yoo et al., 2022), we might expect people with aphasia to show similar effects in an attentional blink paradigm. To our knowledge, this has not been previously studied; however, finding attentional blink differences in people with aphasia would shed light on the complex relationship between attention and language and the underlying mechanisms of its impairment.

### 1.1 Current experiment

Based on the literature and frameworks discussed above, we were interested in exploring the attentional blink in people with aphasia to better understand the role of attention shifting and its impairment in that disorder. Having a better understanding of this relationship has the potential to influence development and refinement of novel treatment methods for aphasia that focus on attention (e.g., Peach et al., 2017, 2019). Therefore, the first aim of the study reported here was to explore attentional blink effects in PWA and age-matched control participants (AMC) using linguistic stimuli. Given the recognition that attention is a domain-general process and that prior research is inconsistent about whether or not the attention impairments in aphasia are language-specific, the second aim of this study focused on attentional blink with nonlinguistic stimuli. Next, given the evidence that some PWA have impairments not only in attention shifting but also in speed of processing, the third aim of this study was to explore speed of processing and its relationship to attention shifting in PWA as compared with AMC. Finally, given the connections found in prior work between rapid attentional processes and word retrieval impairment (Faroqi-Shah and Gehman, 2021; Laganaro et al., 2019), the present study explored the relationship between attentional shifting abilities and severity of word retrieval deficits in PWA to better understand the relationship between attentional processes and lexical access.

With these broad objectives in mind, our research questions and hypotheses were as follows:

1) Do people with aphasia have altered attentional shifting relative to typical adults? We hypothesized that people with aphasia would show impaired attentional shifting, indicating that people with aphasia require more time to overcome attention allocation constraints.
2) Do people with aphasia have different abilities to shift attention for linguistic as compared with nonlinguistic stimuli? We did not have a specific prediction for this question, as the prior literature is inconsistent; however, either finding (similar vs. different performance on the two tasks) would inform our understanding of the relationship between attentional shifting ability and aphasia.
3) Do people with aphasia show distinct patterns in processing speed relative to age-matched controls for both linguistic and nonlinguistic stimuli, and do these patterns correlate to attentional blink performance? We predicted that PWA would show impaired processing speed in both conditions compared to controls, consistent with previous studies. Additionally, we hypothesized that processing speed and attentional blink performance would be correlated, such that individuals with higher accuracy on the processing speed task would also show better performance on the attentional blink task.
4) Is there a relationship between attentional switching deficits and severity of lexical access impairment in people with aphasia? Given the proposed role of attention in lexical retrieval, as discussed above, we hypothesized that PWA with more severe impairments in this language domain would show worse attentional blink performance across both the linguistic and nonlinguistic conditions, suggesting a relationship between attention and language processes.

## 2 Methods

This study used a two-group cross-sectional repeated measures design. The procedures were approved by the Institutional Review Board at San Diego State University.

### 2.1 Participants

Sixteen people with aphasia and nineteen age-matched controls were enrolled. All participants were fluent English speakers. English was the first language for all but three age-matched control participants: one participant acquired English as a second language, and two participants acquired English simultaneously with another language. Participants varied in their self-reported exposure to additional languages other than English; however, as multilingualism was not a primary variable of interest in the present study, it was not inspected further. All participants had normal or corrected-to-normal vision, as assessed using a Tumbling E eye chart at a distance of 10 feet, with aided or unaided visual acuity of at least 20/60.

The participants with aphasia (8 male, 8 female; M_Age_ = 55.19, SD = 14.92) were at least 6 months post-onset of a left-hemisphere stroke, with no significant speech-language or cognitive disorders noted before the stroke and no other neurological disorder, per self-report (see Table 1 for demographic information). The age-matched controls (8 male, 11 female; M_Age_ = 60.05, SD = 14.24) had no history of brain injury, speech-language disorders, or neurological impairment per self-report. A paired *t*-test indicated no significant difference in age between groups (*p* = 0.416).

**Table 1:** Demographics information for participants with aphasia.

Participant	Sex	Race	Languages spoken	Age at testing	Years post-stroke	Years of education	Lesion location
001	M	White	English, Greek, Spanish	64	14	18	L CVA
002	M	White	English	59	7	12	L CVA
003	M	Hispanic	English	66	6	15	L MCA
004	F	ND	English, French	72	9	14	L MCA
005	M	Hispanic	English, Spanish	78	2	18	L frontal lobe
006	F	Black	English	45	3	16	L MCA
007	F	Hispanic	English, Spanish	38	11	14	L CVA
008	M	White	English, Spanish	60	10	20	L CVA
009	F	ND	English	67	10	14	L Temporoparietal
011	M	ND	English	29	7	12	L MCA
012	F	ND	English, Spanish	49	4	16	L Hemisphere (as self-reported)
013	F	White	English	40	6	16	L MCA
014	M	ND	English, Spanish	34	1	14	L medial temporal lobe
016	M	White/Hispanic	English	70	3	20	L Hemisphere (as self-reported)

M = male, F = female, L = left, CVA = cerebrovascular accident, MCA = middle cerebral artery, ND = no data available. Gender data were not collected. Lesion location information as reported by the participant or as available from medical records.

One control participant did not meet the inclusion criteria during pre-testing due to concerns about English language proficiency noted on the Boston Naming Test (Kaplan et al., 1983); thus only data from the Nonlinguistic condition were included in the analysis for that participant (see Data Analysis section). Another control participant’s data were excluded in full due to experimenter error. In addition, two participants with aphasia were discharged from the study after pre-testing; one for scoring above the diagnostic cutoff for aphasia on the Comprehensive Aphasia Test (Swinburn et al., 2004) and one who was found, after testing, to have a history of developmental dyslexia. A total of 14 people with aphasia and 18 age-matched controls participated in the experiment.

### 2.2 Pre-experimental assessment of language and cognition

Both participant groups were assessed using the Boston Naming Test (Kaplan et al., 1983) for picture naming, with inclusion criteria of <50/60 for PWA and >50/60 for AMC. Both groups also completed Raven’s Coloured Progressive Matrices (Raven, 1958) to assess nonverbal reasoning skills, with scores >23/36, and the Symbol Cancellation Test of the Cognitive Linguistic Quick Test (Helm-Estabrooks, 2001) for visual field neglect, with scores >10. The PWA were further assessed using the Comprehensive Aphasia Test (Swinburn et al., 2004) for language abilities; the Elevator Counting and Elevator Counting with Distraction Subtests of the Test of Everyday Attention for sustained and selective attention, adapted for PWA by using a number line (Murray, 2012; Robertson et al., 1994); the Trail Making Test for sustained attention and sequencing (Bowie and Harvey, 2006); the Picture Span Test for verbal short-term memory and working memory (DeDe et al., 2014); and the Pointing Span Test for nonverbal short-term memory and working memory (DeDe et al., 2014) (see Table 2 for assessment scores). The assessments given only to the PWA were for descriptive purposes, not for inclusion or exclusion. Pretests were administered across two sessions for the PWA, to minimize participant fatigue, and in a single session for the control participants.

**Table 2:** Assessment battery scores for participants with aphasia.

PWA	CAT	BNT	RCPM	SCT	TEA subtest 2, 3	TMT A, B	Picture span (forward, backward)	Pointing span (forward, backward)
001	40.5	24	27	12	13, 6	1:10, 3:52	5, 4	3, 2
002	40.75	24	29	0*	7, 4	1:07, 2:57	5, 5	1, 1
003	42.13	25	29	12	7, 3	0:49, 2:15	4, N/A	3, 2
004	39.75	13	32	11	7, 8	1:31, 2:59	4, 3	2, 2
005	54.5	37	21	0*	6, 10	1:22, 9:47	5, 5	3, 2
006	29	0	34	12	7, 9	1:33, 9:45	4, 3	1, 1
007	51.63	48	34	12	7, 5	0:45, 2:12	4, 5	3, 2
008	47.57	46	35	10	6, 10	0:59, 8:02	4, 5	4, 3
009	51.43	43	36	10	7, 5	1:00, 1:51	3, 6	3, 2
011	34.63	20	31	0*	6, 2	1:28, 6:43	4, 3	1, 1
012	53	35	29	11	7, 7	0:26, 1:28	6, 6	3, 3
013	30.63	7	23	9	7, 6	0:40, 3:54	3, 3	1, 1
014	39.25	23	35	12	7, 10	1:16, 3:07	5, 5	2, 2
016	53	49	36	12	6, 9	1:01, 1:11	5, 5	4, 3

CAT = Comprehensive Aphasia Test, mean *t*-scores of all subtests excluding the Gesture Object Use and Arithmetic subtests; BNT = Boston Naming Test; RCPM = Raven’s Coloured Progressive Matrices; SCT = Symbol Cancellation task; TEA = Test of Everyday Attention; TMT = Trail Making Test; ST = span task. *These participants accurately identified all target symbols across the array but also responded to a similar-looking foil; these errors reduced their score despite not having a left neglect.

### 2.3 Equipment

All experimental tasks were completed on a desktop PC running a 64-bit version of Windows 10, connected to a ViewPixx/EEG LCD monitor with 1920 × 1080 resolution. Experiments were run using E-Prime 3.0 (Psychology Software Tools, 2016).

### 2.4 Stimuli

**Figure 1:**
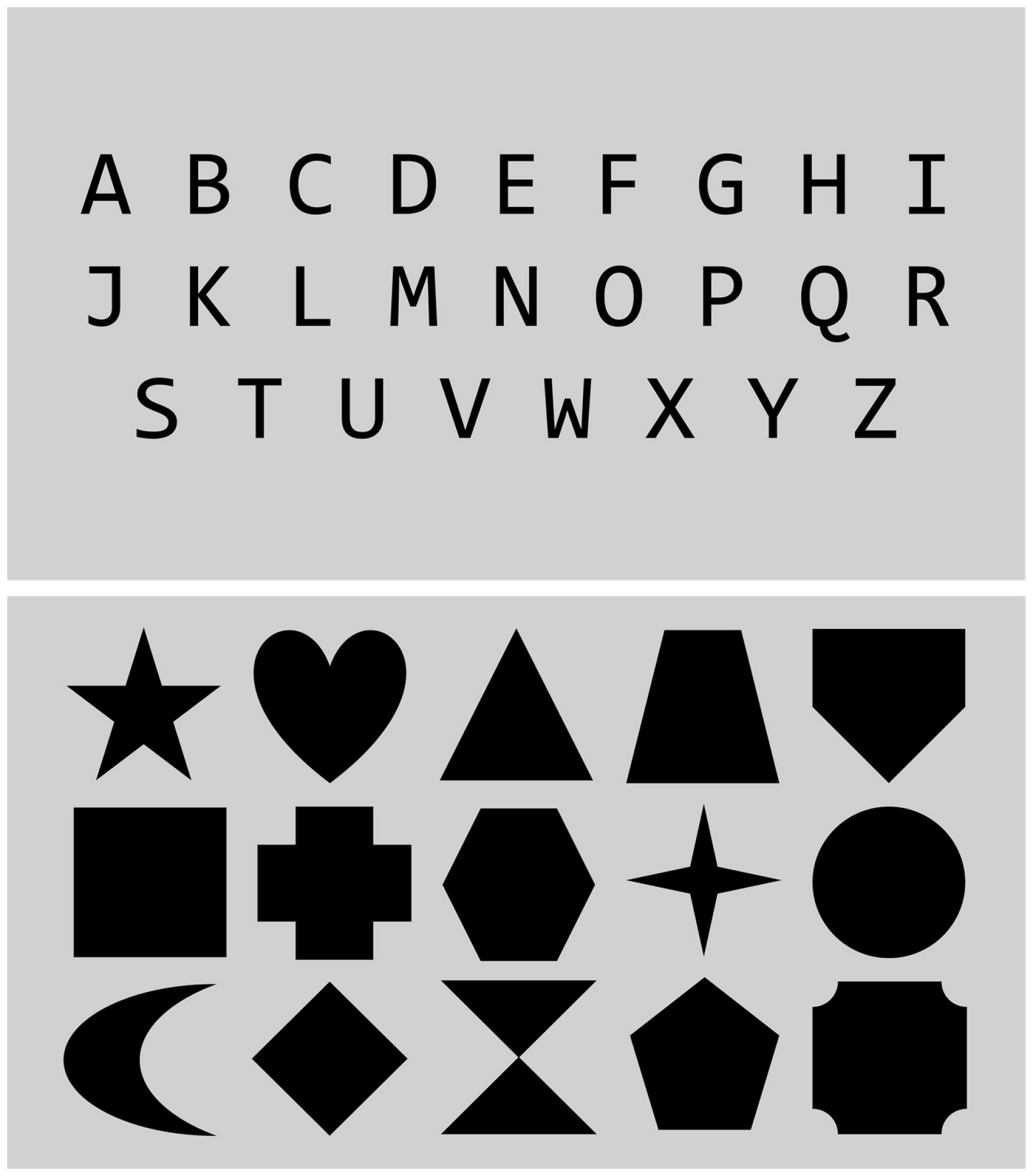
Letter and shape stimuli as presented on the response boards. Though the shapes were not presented with labels throughout the experimental tasks, they were labeled internally with the following naming convention: star, heart, triangle, trapezoid, bookmark (top row, left to right), square, cross, hexagon, compass, circle (middle row, left to right), moon, diamond, hourglass, pentagon, frame (bottom row, left to right).

Stimuli in the Linguistic condition were English capital letters presented in the middle of the computer screen in 70-point Consolas font. Stimuli in the Nonlinguistic condition were black shapes (see Figure 1). Shapes were an average size of approximately 505 × 505 pixels (ranging from 408 × 410 to 513 × 513). All stimuli were black, except for the white T1 stimulus in each trial, and were presented against a light gray background.

### 2.5 Experimental task design

#### 2.5.1 Overview of the RSVP design

An RSVP paradigm, with stimuli flashed sequentially in the center of the screen, was used across all experimental tasks to assess attention shifting and processing speed. The design was modeled after a prior study (Rizzo et al., 2001) that assessed neurotypical adults and adults with chronic focal brain lesions. To assess the attentional blink within the RSVP paradigm, two targets were embedded in a stream of distractor stimuli. In the Linguistic condition, the first target (T1) was a white letter and the second target (T2) was a black X. In the Nonlinguistic condition, T1 was a white shape and T2 was a black triangle.

Standard attentional blink paradigms involve manipulating the stimulus onset asynchronies (SOAs) between T1 and T2 by adjusting the number of distractor stimuli and/or the length of time between them. This creates *lags*, which are the intervals used to determine the time course of the an attentional blink. The total span of time, as measured across lags, at which an attentional blink occurs consistently is defined as the *blink window*. The typical blink window for adults is about 500 ms between T1 and T2 (Raymond et al., 1992); that is, if T2 occurs within 500 ms of T1, it is likely to be missed but if it occurs after a longer interval then it is likely to be detected. Participants with slower processing speed and/or slower attentional shifting require longer intervals of time between T1 and T2 before they begin to notice T2, denoted as a longer attentional blink window (Klein et al., 2011; MacLean and Arnell, 2012; van Leeuwen et al., 2009). In addition, attentional blink is described relative to *blink magnitude*, which is the strength of the attentional blink effect as measured by the degree of decrement in T2 recognition after identifying T1 as compared with T2 recognition when there was no requirement to identify T1, calculated across intervals.

#### 2.5.2 Experimental tasks

**Figure 2:**
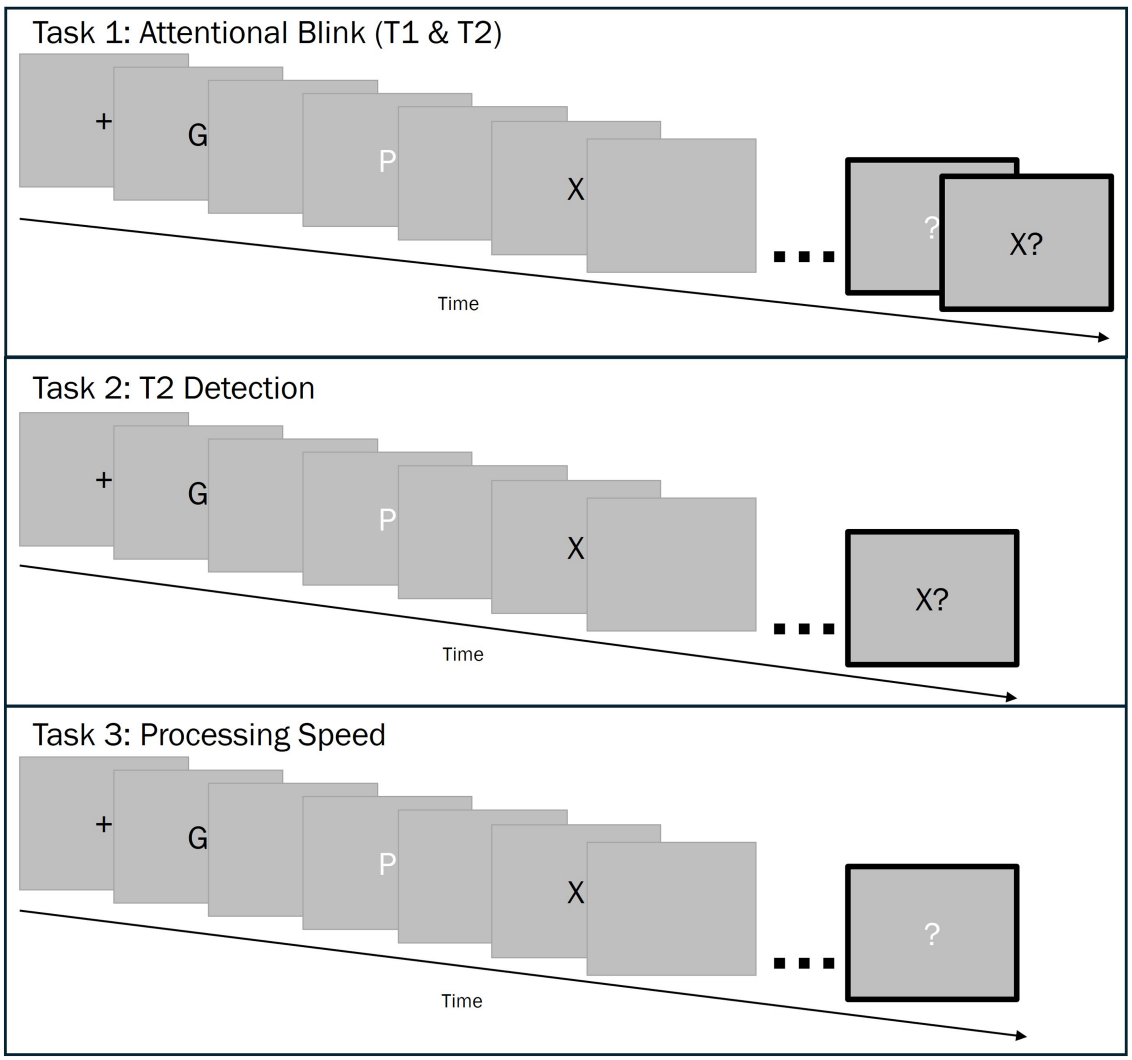
Examples of Tasks 1–3. The last screen(s) in each sequence show(s) what the participant was asked to report: T1 and T2 in Task 1, T2 only in Task 2, and T1 only in Task 3. For Task 3, the presentation speed of the stimuli varied. Only the linguistic condition is depicted; the structure of the nonlinguistic condition was equivalent across tasks using shape stimuli.

In both the Linguistic and Nonlinguistic conditions, there were three experimental tasks (see Figure 2). One Linguistic and one Nonlinguistic stimulus list was constructed and reused across the three tasks in the respective condition. Within a stimulus list, trials either had both T1 and T2 present (i.e., experimental trials) or contained only T1 (i.e., control trials). In experimental trials, the *lag* (i.e., number of distractors inserted between T1 and T2) varied from zero (i.e., lag 1) to five (i.e., lag 6). Furthermore, the location at which the targets occurred in the stream of distractors was also varied to prevent participants from anticipating the onset of T1; T1 was presented either at the beginning (second position), in the middle (ninth position), or toward the end (sixteenth position) of the stream. Each trial contained a total of 23 letters or shapes.

#### 2.5.3 Task 1—Attentional blink task

In Task 1, the attentional blink was assessed using a dual-task paradigm. At the end of each trial, participants were asked to 1) identify T1 (i.e., report which letter/shape was presented in white) and 2) report whether there was a black X/triangle (T2).

**Figure 3:**
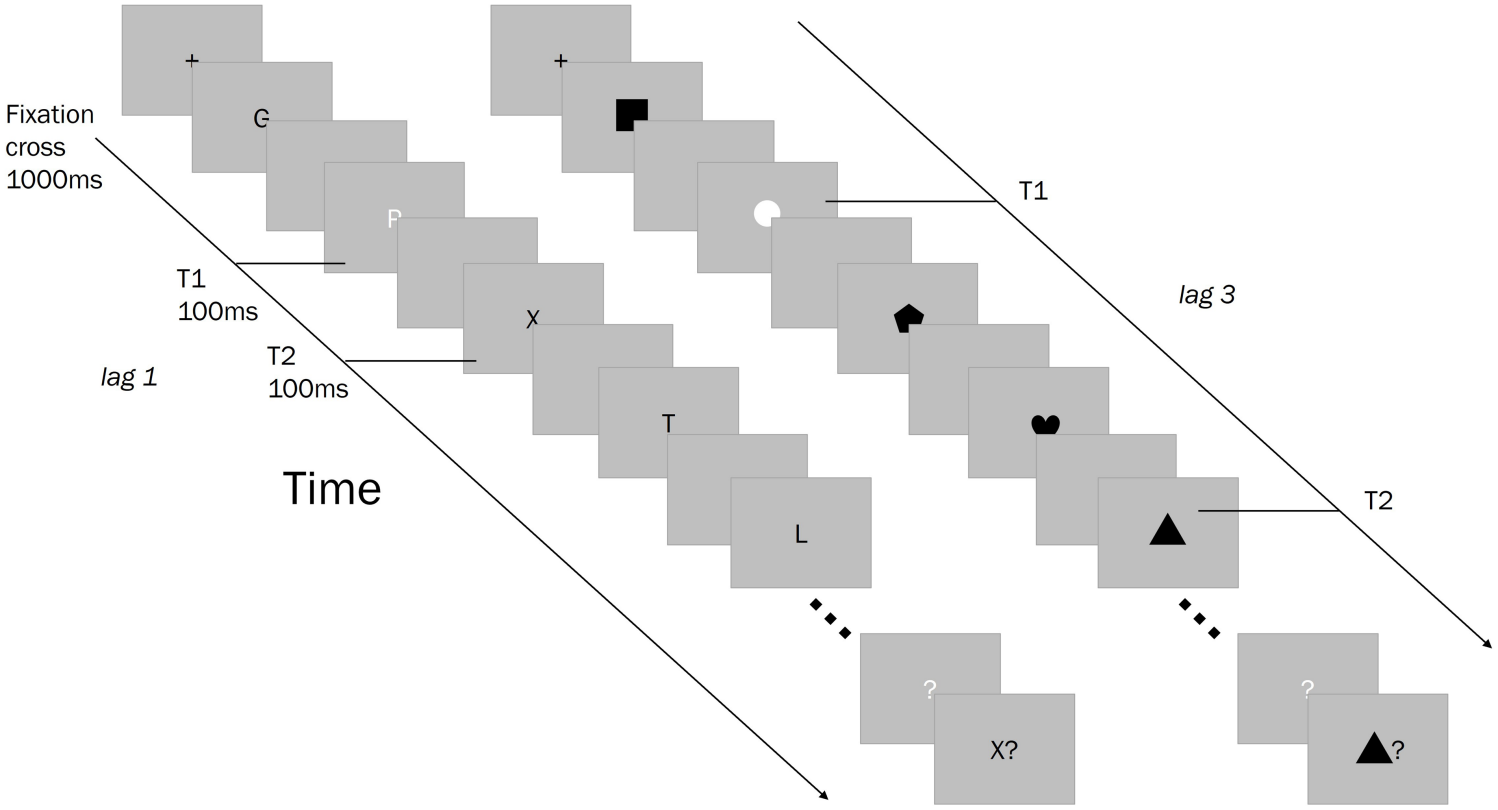
Examples of trials in the linguistic and nonlinguistic conditions. T1 and T2 response screens were presented at the end of each trial. The linguistic example (sequence on the left) shows lag 1; the nonlinguistic example (sequence on the right) shows lag 3.

Each letter/shape in a trial was presented for 100 ms followed by an inter-stimulus interval of 100 ms. At the end of each trial, participants first identified T1 in response to a white question mark. A second screen then displayed a black X/triangle with a question mark, and participants indicated whether T2 had been present (see Figure 3).

In the experimental trials, lag was manipulated by increasing the SOA between T1 and T2 in 200 ms increments. For the shortest interval (i.e., lag 1), T2 followed T1 after 200 ms (zero intervening distractors), and at the longest interval (lag 6), T2 followed T1 after 1,200 ms (5 intervening distractors). Target stimuli were not reused as distractors within the same trial, and distractors never appeared consecutively, although nonconsecutive repetitions within a trial were permitted. For each lag, there were 6 trials, for a total of 36 experimental trials. In addition, there were 36 control trials with only T1 and no T2 present and 6 control trials in which T2 occurred before T1, for a total of 78 trials in each Linguistic and Nonlinguistic Task 1 procedure. Experimental and control trials, as well as trials across all six intervals, were randomized by E-Prime.

#### 2.5.4 Task 2—Single target detection task

In Task 2, a single detection task paradigm was administered using the same stimuli as in Task 1. This task served as a comparison to the dual task, to understand how T2 detection accuracy was affected by the processing costs associated with attention switching in Task 1. In this task, the participant was only asked to report whether there was a black X/triangle (i.e., the presence of T2) and disregarded the white letter/shape. Unlike Task 1, there were 18 experimental trials in which T2 was present and 20 control trials in which T2 was absent, for a total of 38 trials each in the Linguistic and Nonlinguistic conditions. This difference was to reduce the overall testing time, in an effort to minimize fatigue effects. Given the high accuracy rate for this task, the shorter length is not thought to influence the ability to interpret outcomes.

#### 2.5.5 Task 3—Processing speed task

Task 3 measured speed of processing by measuring T1 identification accuracy rates across various stimulus presentation rates. This task was used to determine whether overall processing speed in the Linguistic and Nonlinguistic conditions differed between groups, and to inform our understanding of the relationship between processing speed and attentional blink. Participants were only asked to identify T1 and disregard the black X/triangle.

In Task 3, the stimuli were the same as in Task 1, with 78 trials in each condition, but the trials varied in presentation rates (5, 6.5, 8, 9.5, 11, and 12.5 Hz), such that each stimulus was presented on the screen for 200, 153, 125, 105, 90, or 80 milliseconds, respectively. To keep the number of trials consistent in Task 3 as in Task 1, there were 12 trials using the presentation rates of 6.5 Hz, 9.5 Hz, and 12.5 Hz and 14 trials using the presentation rates of 5 Hz, 8 Hz, and 12.5 Hz. Trials were presented in random order to intermix the presentation rates (see Table 3 for full list of task parameters).

**Table 3:** Task parameters.

Task	Response	Item duration	Number of trials	
			Experimental	Control
Task 1 (dual-task)	Identify T1 and detect T2	100 ms	36	42
Task 2 (T2 detection task)	Detect T2	100 ms	18	20
Task 3 (T1 identification task)	Identify T1	80 ms, 90 ms, 105 ms, 125 ms, 153 ms, 200 ms	36	42

All tasks were completed in the linguistic condition and the nonlinguistic condition. Across all tasks, there were 23 items per trial.

### 2.6 Procedure

Upon successful completion of the pretest sessions, both groups participated in the experimental tasks across two to three sessions, depending on participant fatigue.

Prior to beginning the experimental task in the first experimental session, all participants were assessed for accurate letter/shape recognition by showing them the letters and shapes used in the experiment one at a time and asking them to point to that item on a response board in front of them. The response board was provided for both PWA and control participants throughout all experimental tasks to allow for an accessible response modality; participants were told that they can respond verbally or with pointing for the tasks in the Linguistic condition. For the tasks in the Nonlinguistic condition, all participants were asked to respond only by pointing to the response board in an attempt to minimize the need for verbal coding of the shapes.

Once the experimental tasks began, all participants completed the tasks in the same order: Task 1 (attentional blink task), Task 2 (detection task), and Task 3 (processing speed task); this avoided practice effects on the attentional blink task, which was the primary task of interest. Participants completed all three tasks in one condition (Linguistic or Nonlinguistic) before moving on to the other condition; condition order was randomized across participants. All three tasks were identical in general structure. For each trial in each task protocol, a fixation cross was presented for 1,000 ms and then 23 letters/shapes were presented sequentially in the middle of the screen with a grey background. The presentation order of experimental and control trials was randomized through E-Prime. For all tasks and conditions, participants were positioned about 50 cm from the display. All responses were recorded on paper by the experimenter during the session. A trained reliability judge reviewed the video recordings of the sessions and scored each task, then compared scores with the original responses for agreement.

Before each task, participants were presented with 10 practice trials, during which task instructions were repeated as needed to understand the task. Participants were not informed of their accuracy in either the practice runs or during the experiment. In both the practice and experimental trials, participants were encouraged to take breaks before moving onto the next trial and/or experimental task. The pace of the session was set by the experimenter, who controlled the beginning of each trial.

### 2.7 Data processing and analysis

Analysis began by examining overall response accuracy within each task across groups and conditions. In Task 1, to analyze T2 detection in the context of dual task demands (i.e., the attentional blink effect), trials were discarded if participants incorrectly identified T1; only T1-correct trials were moved forward for analysis. Task 2 and Task 3 data were kept in full for the analysis.

Primary analyses were specified *a priori* to examine group differences in overall task accuracy and attentional blink performance. Two secondary analyses were also planned: the first to examine group differences in processing speed and the second to assess the relationship between processing speed and attentional blink magnitude across individuals.

A post-hoc exploratory analysis was included to understand potential factors contributing to attentional blink performance in the nonlinguistic condition. Furthermore, an additional exploratory analysis was conducted to examine the relationship between attentional blink performance and anomia severity. These analyses were not specified a priori and are therefore interpreted cautiously. Both planned and exploratory analyses are detailed in the following sections.

Table 4 provides a summary of the dependent measures used in each analysis, including the task from which each measure was derived and the interpretation of higher values. Each measure is introduced and its calculation is described in the relevant section below.

**Table 4:** Outcome variables of interest.

Task(s)	Dependent measure	Definition	Interpretation
Task 1 and Task 3	T1 percent accuracy	Mean response accuracy for identifying T1	Higher value = more accurate T1 identification
Task 1 and Task 2	T2 percent accuracy	Mean response accuracy for detecting T2	Higher value = more accurate T2 detection
Task 1 and Task 2	*d′* ratio	Ability to distinguish T2 from distractors at each lag in a dual-task versus single-task, independent of participant’s response bias	Higher value = reduced attentional blink effect (i.e., lower susceptibility to attentional blink and better T2 detection)
Task 1 and Task 2	Blink window	The highest lag at which the *d′* ratio was <0.80	Larger blink window = Longer interval needed between T1 and T2 to overcome attentional blink
Task 1 and Task 2	Blink magnitude	Difference between longest lag (lag 6) and shorter lag (lag 2)	Measure of improvement over lags; trajectory of attentional blink effect. Higher AB magnitude = greater T2 detection recovery across lags
Task 3	Processing speed slope	Slope of T1 accuracy across presentation speeds	Greater slope = greater change in T1 accuracy across increasing presentation speeds

#### 2.7.1 Attentional blink (Tasks 1 and 2)

Consistent with prior research (Rizzo et al., 2001), we applied Signal Detection Theory to explore attentional blink effects, using two related measures: *d′* and *d′ ratio*. The *d′* calculations captured response accuracy while accounting for response bias. In both Task 1 (dual task) and Task 2 (single target detection task), *d′* was calculated for each condition, task, and (in Task 1) lag, by first obtaining the Hit Rate (i.e., accurate detection of T2 when it was present) and the False Alarm Rate (i.e., T2 reported when it was not present). The *d′* value was then calculated by taking the z-scores of the rates using this formula:


$$d^{\prime }=z(\mathrm{Hit}\;\text{Rate})–z(\text{False Alarm Rate})$$

The *d′* ratio was then calculated to compare the *d′* from the single detection task (Task 2) to the *d′* from the dual task (Task 1). This involved dividing *d′* from Task 1 by *d*′ from Task 2 as shown in the following formula:


$$\frac{\left(z(\text{Task}\;1\;\mathrm{Hit}\;\text{Rate})-z(\text{Task}\;1\;\text{False Alarm Rate})\right)}{\left(z(\text{Task}\;2\;\mathrm{Hit}\;\text{Rate})-z(\text{Task}\;2\;\text{False Alarm Rate})\right)}$$

The *d′* ratio captures the change in sensitivity to T2 between the single-task and dual-task conditions, thus capturing the effect of attentional switching demands in Task 1.

From the *d′* ratio for each lag, the length of the blink window was determined, defined as the highest lag at which the *d′* ratio was <0.80 (Rizzo et al., 2001). Therefore, the presence of a blink across a greater number of lags indicated a longer blink window (for further explanation, see Table 4). Data visualization and subsequent analyses were conducted using R, version 4.5.2 (R Core Team, 2022) with the package ggplot2, version 4.0.1 (Wickham, 2016).

#### 2.7.2 Processing speed (Task 3)

For Task 3, to understand if and how processing speed is impacted in people with aphasia compared to controls, T1 identification accuracy was analyzed across stimulus conditions and stimulus presentation speeds (i.e., frequency; see Table 4 for interpretation of values). Analysis was conducted using generalized linear mixed effects models, which are suited for response data with a binary outcome (Jaeger, 2008). The fixed effects of Frequency, Group, and Condition were explored, with Participant included as a random effect. Models were run via the *lme4* package in R, version 1.1.37 (Bates et al., 2015).

#### 2.7.3 Relationship between attentional blink magnitude and processing speed

A correlation analysis was conducted to determine how individual differences in processing speed impacted attentional blink performance (Rizzo et al., 2001). To do so, the *d′* ratio was calculated for each participant at each lag. A loglinear approach (Hautus, 1995; Stanislaw and Todorov, 1999) was used as a corrective measure to address the extreme values (i.e., 0 or 1) in the data, adding 0.5 to both the number of hits and the number of false alarms, and adding 1 to both the number of signal trials and the number of noise trials, before calculating the hit and false alarm rates. The participant-level *d′* ratios for each lag were then transformed into a single attentional blink magnitude (MacLean and Arnell, 2012; Zhou et al., 2020) to allow comparison of the overall trajectory of attentional blink as a single measure (across lags) with processing speed. Each participant’s blink magnitude was calculated by subtracting their *d′* ratio at lag 2 from their *d′* ratio at lag 6. A higher blink magnitude is therefore associated with a greater attentional blink improvement (i.e., greater T2 detection) across increasing intervals.

For the processing speed data, average T1 accuracy for each participant at each frequency was calculated from the raw accuracy data in Task 3. Then a slope was calculated across the 6 frequencies to capture each participant’s processing speed. Subsequently, the processing speed slopes and the blink magnitudes were compared using Spearman’s correlations (see Table 4 for full list of outcome measures and their interpretations).

#### 2.7.4 Relationship between anomia severity and attentional blink magnitude

To understand how aphasic language impairments may relate to difficulties in attention shifting, post-hoc exploratory analyses were performed using the PWA individual blink magnitudes from the Linguistic condition. Anomia severity was measured using performance on the Boston Naming Test (BNT). The relationship between these two sets of scores were then analyzed using a linear regression model.

In addition, in recognition that cognitive mechanisms interact, we also performed a post-hoc exploratory path analysis. This was motivated by, and based on, prior data showing explanatory mediation effects for the attentional blink in which simple effects were absent but mediated effects were found, driven by T1 identification accuracy independent of T2 accuracy (Zhou et al., 2020). Based on this finding, and to explore potential explanatory mediation effects between naming ability and attentional blink in our data set, post-hoc exploratory analyses were conducted to further understand the relationship between BNT scores and blink magnitudes using a mediation model via the *lavaan* (v 0.6-21) package in R (R Core Team, 2022; Rosseel, 2012).

## 3 Results

Accuracy data from 14 people with aphasia and 18 age-matched controls were included in the analyses. One of the control participants contributed data only to the Nonlinguistic condition because they did not meet the BNT score criterion, as described above. Thus, the Nonlinguistic condition included data from 32 participants, whereas the Linguistic condition included data from 31 participants.

### 3.1 Reliability

Throughout the six experimental tasks, an experimenter recorded responses with pen and paper. Inter-rater reliability for all the responses was performed by two trained research assistants, with greater than 98% agreement on all tasks (Task 1 = 98.24%, Task 2 = 99.9965%, Task 3 = 99.9852%).

### 3.2 Task accuracy

**Figure 4:**
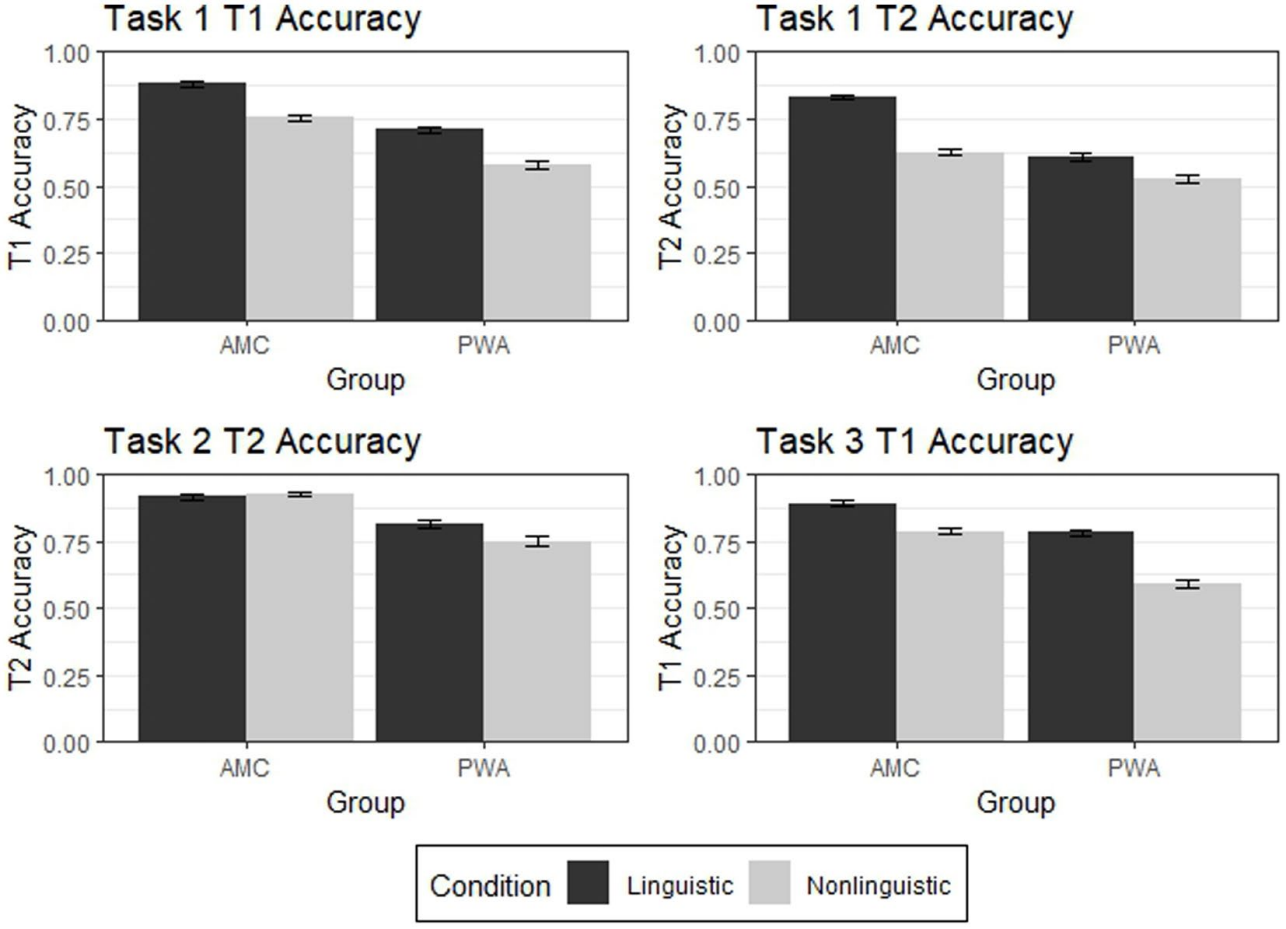
Overall T1 and T2 accuracy across tasks. Only the mean percent accuracy is plotted here.

Mean accuracy for AMC ranged from 62.59 to 92.46% across all tasks, while mean accuracy for PWA ranged from 52.84 to 81.32% (see Figure 4). Generalized linear mixed effects models revealed significant main effects of Condition and Group across all three tasks (see Table 5).

**Table 5:** Generalized linear mixed effects model results for task accuracy.

Task	Measure	Fixed effects	Estimate	Standard error	*Z*	*p*
Task 1	T1 Accuracy	Intercept	2.21	0.24	9.31	<0.001***
		Group	−1.16	0.35	−3.36	<0.001***
		Condition	−0.80	0.08	−10.54	<0.001***
	T2 Accuracy	Intercept	0.38	0.10	13.83	<0.001***
		Group	−0.76	0.14	−5.55	<0.001***
		Condition	−0.69	0.06	−10.64	<0.001***
Task 2	T2 Accuracy	Intercept	3.02	0.29	10.47	<0.001***
		Group	−1.40	0.39	−3.56	<0.001***
		Condition	−0.34	0.13	−2.63	0.01**
Task 3	T1 Accuracy	Intercept	2.39	0.20	12.03	<0.001***
		Group	−1.04	0.28	−3.67	<0.001***
		Intercept	−0.91	0.08	−11.44	<0.001***

		Random effects	Variance	Standard deviation
Task 1	T1 Accuracy	Participant (intercept)	0.89	0.94
	T2 Accuracy	Participant (intercept)	0.11	0.34
Task 2	T2 Accuracy	Participant (intercept)	1.02	1.01
Task 3	T1 Accuracy	Participant (intercept)	0.57	0.76

The same model format was applied for all four models. R model equation: Accuracy ~ Group + Condition + (1 | Participant). **p < 0.01. ***p < 0.001.

### 3.3 Attentional blink group analysis

**Figure 5:**
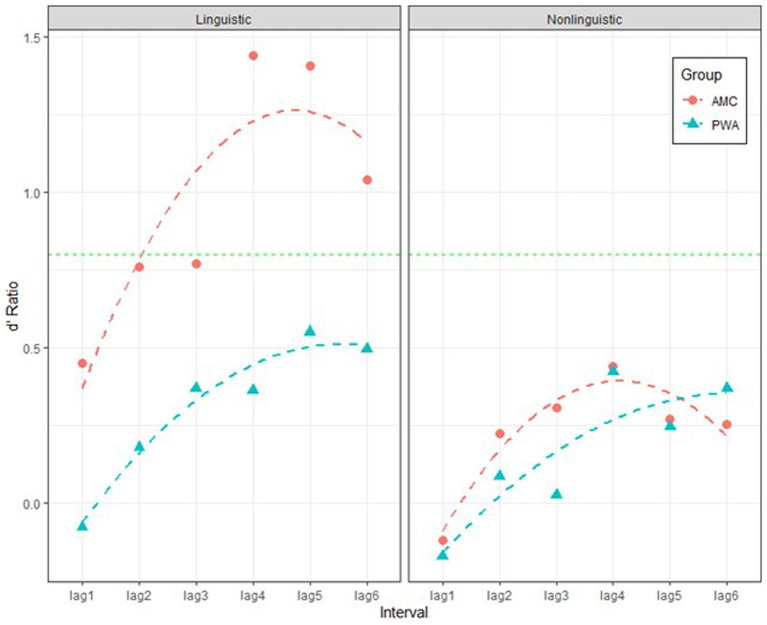
Attentional blink effects.

In the Linguistic condition, the age-matched controls had larger *d′* ratios (i.e., fewer blinks) as the intervals between T1 and T2 increased (see Figure 5). Using a *d′* ratio lower than 0.8 as the threshold defining the presence of an attentional blink (Rizzo et al., 2001), the age-matched controls showed a blink window from lags 1 through 3. The people with aphasia also showed an increase in *d′* ratio as the intervals increased, though at a slower rate than controls. Using the 0.8 threshold, the PWA showed a blink at all lags. The AMC demonstrated higher *d′* ratios across all intervals compared to PWA, indicating that AMC were less likely than PWA to show attentional blinks across intervals.

In the Nonlinguistic condition, unlike in the Linguistic condition, the AMC and PWA demonstrated similar *d′* ratios. Both groups performed under the 0.8 threshold across all lags, indicating that neither group reliably identified T2 at any lag; that is, both groups were likely to show attentional blinks even as intervals increased. A two-way Analysis of Variance (ANOVA) was then used to assess the relationship between Group and Condition. The ANOVA revealed a significant interaction between Group and Condition (*F*_1,20_ = 7.35, *p* = 0.01), which a subsequent Tukey’s HSD test indicated was driven by the difference between groups in the Linguistic condition in contrast to the lack of difference across conditions for AMC. In other words, the PWA performed significantly below the AMC in the Linguistic condition (i.e., had larger attentional blinks and a larger blink window), but this difference disappeared on the Nonlinguistic task, in which the AMC performed significantly below their performance in the Linguistic condition. PWA did not perform significantly differently across conditions.

### 3.4 Processing speed analysis

**Figure 6:**
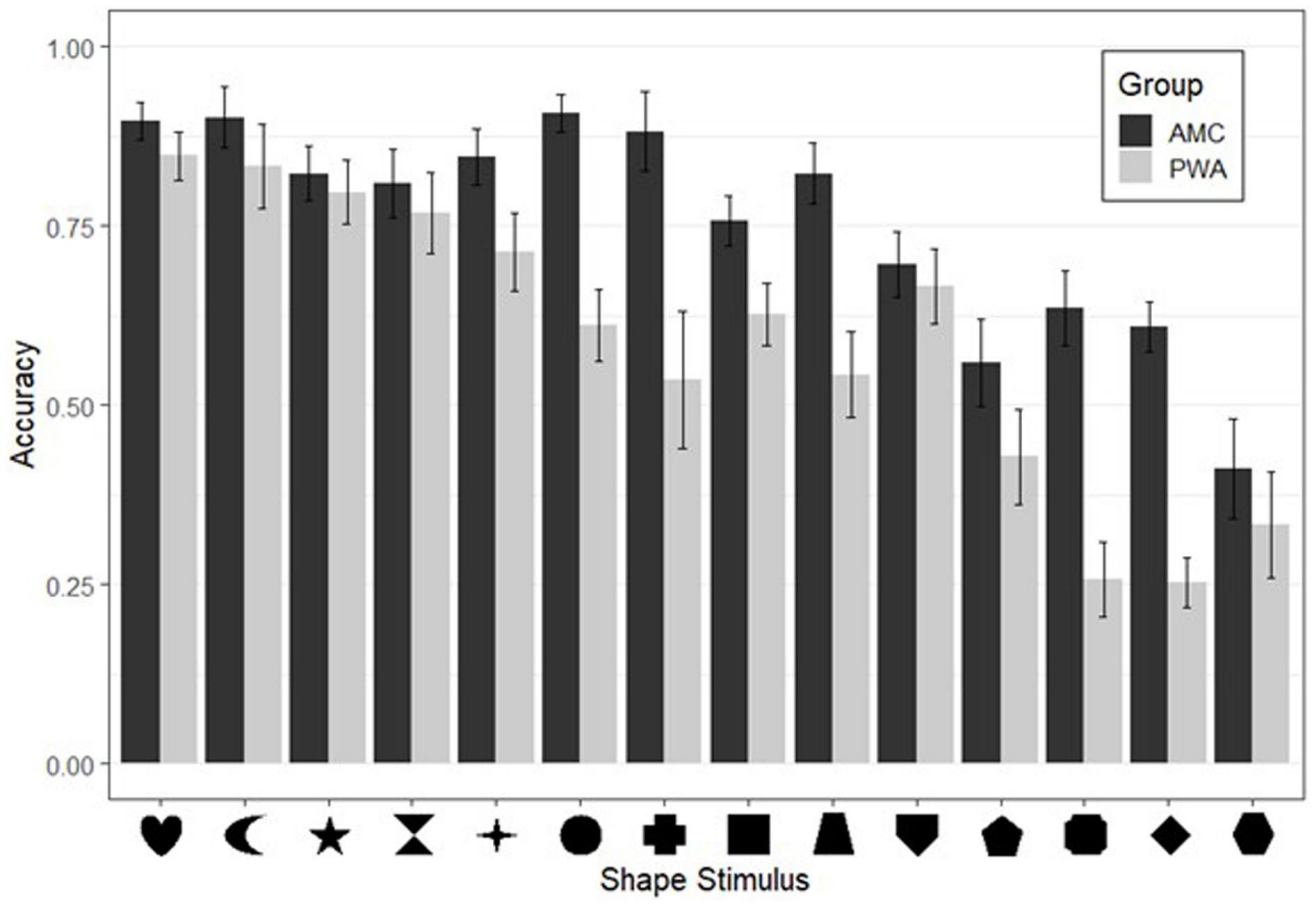
Item identification accuracy for nonlinguistic condition in Task 1.

Task 3 data were analyzed to understand how processing speed varied across Frequency, Group, and Condition (see Figure 6). Type III Wald Chi-square tests revealed significant main effects of Frequency [*χ*^2^ (5) = 309.14, *p* < 0.05], significant main effects of Group [*χ*^2^ (1) = 13.38, *p* < 0.05], and significant main effects of Condition [*χ*^2^ (1) = 141.33, *p* < 0.05; see Table 6]. A subsequent model including interactions revealed a significant Frequency x Group x Condition interaction [*χ*^2^ (5) = 13.56, *p* = 0.02].

**Table 6:** Generalized linear mixed effects model results with multiple comparisons for shape stimuli.

Fixed effects	Estimate	Standard error	*Z*	*p*
Intercept	2.86	0.33	8.70	<0.001***
Group	−1.11	0.37	−2.97	0.003*
Heart vs. Bookmark	−1.42	0.27	−5.20	<0.001***
Heart vs. Circle	−0.85	0.27	−3.11	0.002**
Heart vs. Compass	−0.76	0.30	−2.55	0.01*
Heart vs. Cross	−1.17	0.38	−3.10	0.002**
Heart vs. Diamond	−2.61	0.24	−10.67	<0.001***
Heart vs. Frame	−2.53	0.278	−9.12	<0.001***
Heart vs. Hexagon	−2.98	0.32	−9.32	<0.001***
Heart vs. Hourglass	−0.74	0.32	−2.32	0.02*
Heart vs. Moon	−0.04	0.40	−0.11	0.91
Heart vs. Pentagon	−2.36	0.29	−8.11	<0.001***
Heart vs. Square	−1.33	0.25	−5.21	<0.001***
Heart vs. Star	−0.58	0.29	−1.97	0.049*
Heart vs. Trapezoid	−1.34	0.28	−4.70	<0.001***

Random effects	Variance	Standard deviation
Participant (intercept)	0.98	0.99

R model equation: Accuracy ~ Group + Shape Stimulus + (1 | Participant). *p < 0.05. **p < 0.01. ***p < 0.001.

### 3.5 Processing speed and attentional blink performance correlation

**Figure 7:**
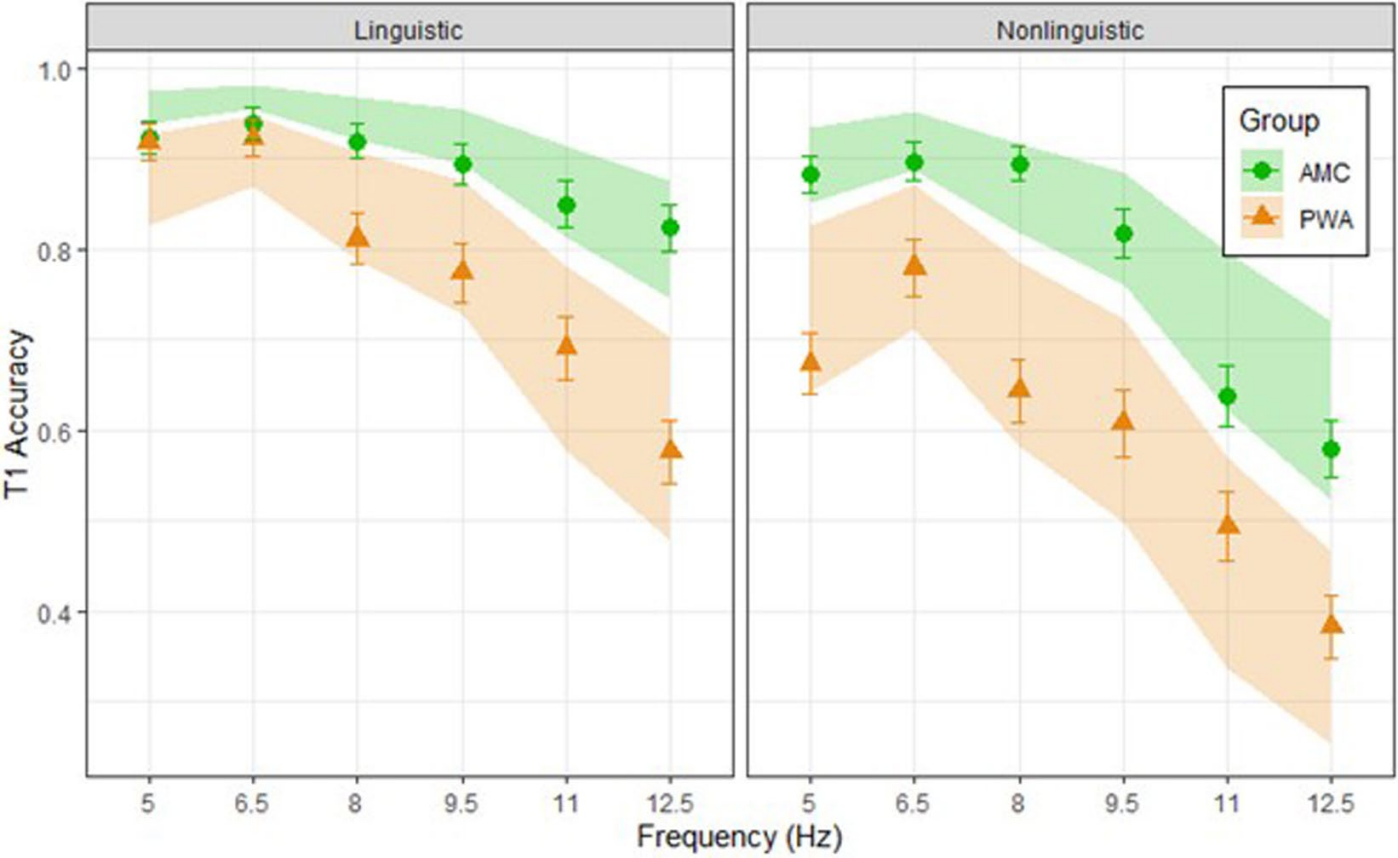
Observed accuracy and model prediction in processing speed task. The observed T1 accuracy data from Task 3, with the frequency of presentation of the stimuli on the x-axis and the accuracy on the y-axis. The AMC are plotted in green and PWA are plotted in orange. The predicted probabilities as determined by the model are plotted in the ribbons, with the thickness indicating confidence intervals.

A Spearman correlation exploring the relationship between processing speed and attentional blink magnitudes was not significant with the groups combined (*R* = −0.04, *p* = 0.84). Correlations within each group were not significant for either the AMC (*R* = −0.30, *p* = 0.27) or the PWA (*R* = −0.44, *p* = 0.11; Figure 7).

### 3.6 Post-hoc attentional blink item analysis

Post-hoc analyses explored the significant decrease in performance for AMCs in the Nonlinguistic condition. Specifically, the shape stimuli were examined to determine if the shapes varied in terms of difficulty. Generalized linear mixed effects models were used to explore the fixed effects of Group and Item (i.e., shape stimuli) on T1 identification accuracy during Task 1, with Participant included as a random effect. These analyses were intended to explore overall item-level variability; therefore, we did not exhaustively compare all pairwise differences between stimuli. To facilitate interpretation, the shape with the highest accuracy rate (the heart) was used as the reference level. Wald Chi-square tests revealed a significant main effect of Group [*χ*^2^ (1) = 8.79, *p* = 0.003] and main effect of Shape [*χ*^2^(13) = 279.87, *p* < 0.001] on the likelihood of T1 accuracy (see Table 7), indicating significant differences in accuracy across shape stimuli. An additional model was implemented to explore whether there was an interaction between group performance and item-level T1 identification accuracy. There was a significant interaction effect between Group and Shape stimuli [*χ*^2^ (13) = 40.15, *p* < 0.001], and model fit was significantly improved [*χ*^2^ (13) = 41.85, *p* < 0.001].

**Table 7:** Generalized linear mixed effects model results with multiple comparisons for processing speed.

Fixed effects	Estimate	Standard error	*Z*	*p*
Intercept	3.19	0.24	13.35	<0.001***
Group	−1.13	0.31	−3.66	<0.001***
Condition	−0.99	0.08	−11.89	<0.001***
5 Hz vs. 6.5 Hz	0.34	0.16	2.11	0.035*
5 Hz vs. 8 Hz	−0.26	0.14	−1.79	0.07
5 Hz vs. 9.5 Hz	−0.59	0.14	−4.13	<0.001***
5 Hz vs. 11 Hz	−1.27	0.14	−9.18	<0.001***
5 Hz vs. 12.5 Hz	−1.67	0.13	−12.60	<0.001***

Random effects	Variance	Standard deviation
Participant (intercept)	0.68	0.83

R model equation: Accuracy ~ Frequency + Group + Condition + (1 | Participant). *p < 0.05. **p < 0.01. ***p < 0.001.

### 3.7 Post-hoc anomia severity and attentional blink analysis

A linear regression explored whether attentional shifting ability, as indexed by attentional blink effects, predicts naming ability, as indexed by BNT score, for PWA. This analysis used the blink magnitudes in the Linguistic condition for each PWA. The model was not significant (*F*_(1, 12)_ = 1.59, *p* = 0.23, *R*^2^ = 0.12), suggesting that AB magnitude did not predict BNT score [*β* = 9.22, *t*(12) = 1.26, *p* = 0.23; Table 8].

**Table 8:** Path analysis results.

Antecedent	T1 Accuracy				Blink magnitude			
	Path	*B*	*SE*	*p*	Path	*B*	*SE*	*p*
constant	*i_M_*	0.019	0.013	0.132	*i_Y_*	0.124	0.035	0.000
BNT Score	*a*	−0.001	0.002	0.702	*c’*	0.015	0.006	0.012*
T1 Accuracy					*b*	2.742	1.564	0.080
	*R*^2^ = 0.006				*R*^2^ = 0.595			
Indirect effect: *B* = −0.002, *SE* = 0.007, *p* = 0.754								

*p < 0.05.

**Figure 8:**
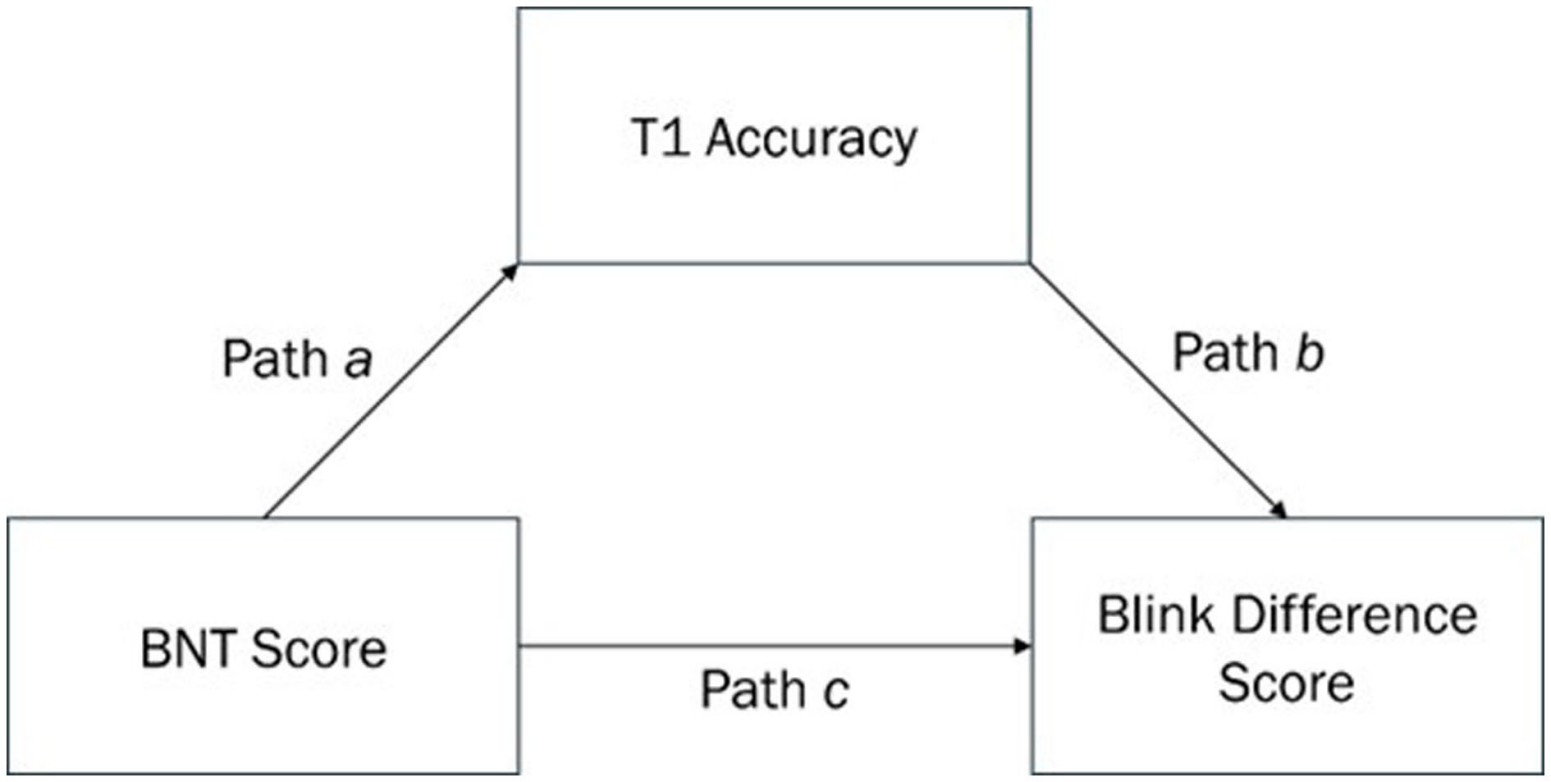
Path analysis diagram.

We then explored whether including T1 accuracy, a measure thought to index verbal short-term memory, altered the relationship between attentional blink magnitude and BNT scores by using a path analysis/mediation model that examined the relations between all three of these factors. Specifically, the relationships examined were the direct effect of blink magnitude on T1 accuracy (path *a*), the direct effect of T1 accuracy on BNT scores (path *b*), the direct effect of blink magnitude on BNT scores (path *c*), and the indirect effect of blink magnitude on BNT scores with T1 accuracy as a mediator (see Figure 8).

The model was just-identified, so global model fit indices are not interpreted (Streiner, 2005). With respect to the relations specified within the model, the effect of blink magnitude on T1 accuracy (path a) was significant (*B* = 0.17, *SE* = 0.08, *p* = 0.03), indicating an association between blink performance and T1 accuracy (see Table 6). The path from T1 accuracy to BNT score (path b) was not significant (*B* = −57.94, *SE* = 66.59, *p* = 0.38), suggesting no relationship between T1 accuracy and word retrieval deficits. In contrast to the initial linear regression model, however, the direct path from blink magnitude to BNT score (path c) was statistically significant (*B* = 18.91, *SE* = 8.97, *p* = 0.04), indicating that PWA with higher blink magnitudes also showed higher BNT scores. Consistent with these results, the indirect effect of blink magnitudes on BNT scores (i.e., the mediation effect) via T1 accuracy was not significant (*B* = −9.70, *SE* = 6.28, *p* = 0.12).

## 4 Discussion

This study used an attentional blink paradigm to investigate attention shifting in people with aphasia and age-matched controls for both linguistic and nonlinguistic stimuli. In the linguistic condition, letter stimuli were presented with two targets embedded in a rapidly presented visual stream with varying inter-stimulus intervals and participants were asked to report on both targets. The Nonlinguistic condition used shape stimuli instead of letters for the same task. As predicted, differences in performance were observed between the groups and conditions, though the pattern that emerged was unexpected. In addition, to explore potential factors contributing to the attentional blink effects, speed of processing was assessed within the same protocol through modulating the presentation speed. An additional mediation analysis was conducted to explore the relationship between attentional blink performance and word retrieval ability.

### 4.1 Attentional shifting and linguistic processing

In the linguistic condition, the age-matched control group was found to successfully disengage from T1 to process T2 in a fairly typical time course, demonstrated by an attentional blink (i.e., missing the presence of T2) across the three shortest intervals but reliable identification of T2 at longer intervals. In contrast, the PWA group showed an attentional blink effect across all six intervals assessed, suggesting a poor ability to disengage from T1 and shift attention adequately to identify the presence of T2 following T1. These differences in the attentional blink window between groups suggest an impairment in attentional shifting in PWA compared to control participants for the linguistic stimuli.

These findings expand upon a prior study (Rizzo et al., 2001) that demonstrated that participants with focal cerebral lesions had significantly impaired attentional blink magnitudes and blink lengths/windows. In that study, however, the focal lesion group surpassed the blink threshold (i.e., no longer showed an attentional blink) at longer intervals (i.e., SOA of 800–1,200 ms), indicating that they eventually successfully shifted their attention. In the present study, the PWA performed below the blink threshold at all intervals assessed. This finding aligns with our hypothesis that the PWA would demonstrate difficulty with shifting attention in the linguistic condition due to the distinct language impairments associated with the left hemisphere in PWA. More broadly, these results are consistent with prior studies suggesting that some PWA exhibit impairments in attention shifting (Chiou and Kennedy, 2009; Kuptsova et al., 2023), as well as broader attentional difficulties reported in this population (Heuer and Hallowell, 2015; Hunting-Pompon et al., 2011; LaCroix et al., 2021; Murray, 2012). The findings in the present study extend this literature by identifying a similar impairment in attentional shifting in the context of a task that is rapid and temporally constrained.

### 4.2 Attentional shifting and nonlinguistic processing

In the Nonlinguistic condition, the PWA did not differ significantly from their performance in the Linguistic condition; they showed attentional blinks at all intervals. Furthermore, and unexpectedly, the AMC demonstrated decreased performance in the Nonlinguistic condition compared to the Linguistic condition, with attentional blinks noted at all intervals and no significant differences from the PWA. In addition, when comparing the AMC’s accuracy data across all tasks, it is apparent that the Nonlinguistic condition overall resulted in decreased accuracy, particularly in Task 1 and Task 3. The finding that AMC participants failed to successfully shift attention in the Nonlinguistic condition, together with the difficulty observed when reporting T1 alone in Task 3, suggests that the task may have imposed demands beyond the intended measure of nonlinguistic attentional shifting.

The timing parameters used in the present study were the same as those used by Rizzo et al. (2001), as the letter-based task was intended to replicate that paradigm. However, the nonlinguistic condition introduced a new stimulus set: shape stimuli rather than letters. Although attentional blink paradigms have used a range of stimuli, including letters, objects, faces, and colors, stimulus characteristics can influence the magnitude and time course of the attentional blink (e.g., Landau and Bentin, 2008; Raymond, 2003). In the present study, the shape stimuli varied in visual complexity, discriminability, and label familiarity, which could have increased demands on visual encoding, working memory, or response selection.

This interpretation may help explain why the AMC group did not show the expected attentional blink trajectory across longer T1–T2 intervals. Specifically, the intended analysis was predicated on the assumption that the AMC participants would show a typical attentional blink pattern across lags, characterized by attentional blink effects at shorter T1-T2 intervals, but would no longer show blink effects at longer intervals. This pattern would have provided a neurotypical comparator for the PWA data and allowed us to examine whether nonlinguistic attentional shifting between PWA and neurotypical adults. However, this assumption was not met, as the AMC group showed attentional blinks across all T1-T2 intervals. This result was surprising and suggests that the task that was used did not cleanly isolate attentional shifting as expected and did not provide a clear baseline against which to interpret the PWA data. We therefore do not interpret the PWA results from this task as evidence for or against impaired nonlinguistic attentional shifting in aphasia.

It is not clear why the nonlinguistic task did not yield the expected results for the AMC group. One possibility is that, although the T1–T2 intervals matched those used by Rizzo et al. (2001), the use of shape stimuli may have required more time for visual encoding or response selection than the letter stimuli used in the original paradigm, so the intervals may not have been sufficient to capture the intended effects; longer intervals may yielded different results. Another potential explanation for these findings is that shapes differed in terms of visual complexity or discriminability, affecting the ease with which they were processed and encoded (Dai et al., 2022; Sun and Firestone, 2021). This possibility is supported by the post-hoc item analysis for Task 1, which revealed clear differences across shape stimuli in the accuracy with which T1 was identified. A third potential explanation is that, even though the response modality was visual/pointing rather than naming, participants attempted to verbally encode the shape stimuli (i.e., name them to assist in remembering them) but that the various shape stimuli differed in how easy they were to name, making some items easier to encode and recall then others. For example, the heart, moon, circle, and cross stimuli are highly concrete and familiar and have readily available labels, which may have contributed to their highly accurate processing by the AMC group relative to many of the other shapes. At the same time, though, those patterns did not hold up for the PWA, which complicates the ability to accept this interpretation. Overall, the findings suggest that item-level characteristics, hypothesized to be related to visual complexity and/or label familiarity, may have influenced T1 identification accuracy for the nonlinguistic task and, therefore, attentional blink outcomes. Future work with larger samples and stimulus-level analyses will be needed to determine which stimulus characteristics may need to be better controlled, or to devise other tasks that eliminate the need for, or influence of, these stimulus characteristics to provide a clear understanding of the status of nonlinguistic attentional blink in aphasia.

Regardless of the reason for difficulty with the Nonlinguistic version of Task 1, having a low T1 accuracy rate resulted in being unable to calculate attentional blink effects for most of these items. In addition, these results suggest that performance on this task reflected more cognitive processing challenges than just attentional shifting, likely leading to greater demands on working memory to successfully complete the task. As such, these results cannot be confidently interpreted and cannot inform our understanding of nonlinguistic attentional shifting in aphasia. At the same time, however, these results highlight considerations for task design if this line of inquiry is to be pursued. Specifically, future studies should establish ways to engage nonlinguistic attentional shifting in ways that do not add to the cognitive load of the task, such as assuring similar visual salience of all stimuli and/or reducing the set of items needing to be reported to ease the processing/working memory load of the task.

### 4.3 Processing speed

In addition to the attentional blink, given that the study on which this protocol was based (Rizzo et al., 2001, discussed further below) found some evidence for a relationship between processing speed and attentional blink window, we also explored processing speed in PWA and AMC using the same set of stimuli presented at varying rates. As expected, both groups demonstrated decreased task accuracy as the frequency of the stimulus presentation increased, but there was a significant difference between groups in both conditions, with PWA demonstrating overall lower accuracy across presentation rates. Performance in the Nonlinguistic condition was significantly worse than the Linguistic condition overall, which again suggests increased difficulty processing the shape stimuli compared to letter stimuli.

Though PWA as a group demonstrated impaired attentional blink and slower processing speeds compared to control participants, we did not observe statistically significant correlations between individual differences in attentional blink performance and processing speed in the present sample. This finding is broadly consistent with prior data from neurotypical control participants and those with focal acquired brain lesions (Rizzo et al., 2001), showing no significant between-group or within-group correlations between individual processing speed and attentional blink performance. Interestingly, Rizzo et al. found a significant correlation between processing speed and the length of the attentional blink window, but only when control participants and patients with lesions were combined into a single group. Taken together, these findings may suggest that, although PWA demonstrate overall processing speed impairments, the differences noted here in attentional shifting may not simply reflect an overall reduction in processing speed.

The absence of statistically significant correlations between processing speed and attentional blink performance in the present sample is also consistent with prior work that has similarly failed to identify a relationship in neurotypical adults (e.g., Arnell et al., 2006; Badcock et al., 2008). At the same time, however, others have found evidence of an indirect connection, such that processing speed can predict one’s ability to accurately report targets, which consequently affects the ability to identify attentional blinks (Willems and Martens, 2016). In addition, processing speed has been found to be associated with attentional blink performance when the target and distractors were visually similar; when the distractors had low visual similarity to the target, there was no longer an association (Visser and Ohan, 2012). Thus, although we did not identify a statistically significant association in this study, it is possible that processing speed may not be a factor in attentional shifting per se, but may be a factor in predicting target accuracy, which then affects attentional blink. Future studies with larger sample sizes and protocols that are designed specifically to disambiguate these factors are needed to fully understand these relationships. However, because the observed correlations in the present study were not negligible and the sample size was limited, these findings should not be interpreted as definitive evidence that processing speed and attentional blink performance are unrelated.

### 4.4 Word retrieval and attentional blink

Finally, given prior evidence of impaired attention shifting in PWA, we explored whether anomia severity was associated with attention shifting. Finding this association would lend support to the theories proposing that attention plays a foundational role in language deficits and would specifically highlight the role of attention shifting, which has not been studied in this population. The exploratory path analysis suggested that higher blink magnitude, as indexed by greater differences in T2 detection across lags, was associated with milder word retrieval deficits when T1 accuracy was controlled. Because the blink magnitude was calculated by comparing performance at a longer lag to performance at a shorter lag, this association may indicate that individuals with milder word retrieval deficits showed greater improvements in T2 detection from the short lag to the long lag. In contrast participants with more severe word retrieval deficits showed a more sustained deficit in detecting T2, resulting in smaller differences across lags.

We propose that the relationship with T1 accuracy may reflect the dependence of attentional blink measures on successful T1 identification. To effectively shift attention from T1 to T2, and thereby avoid an attentional blink, one must be able to rapidly consolidate T1 into memory storage while inhibiting processing of distractors, and then process T2 (Zhou et al., 2020). Thus, in our analysis, although there was no evidence of a significant relationship between lexical retrieval and T1 accuracy, the T1 accuracy variable may be indexing memory consolidation skills (Minkina et al., 2018). By controlling for T1 performance in our analysis, we are largely removing the effect of short-term memory in the attentional blink task and uniquely isolating the relationship between attention shifting and anomia severity. Given the small sample size and exploratory nature of this analysis; however, these findings are preliminary and should be interpreted cautiously.

Taken together, these preliminary findings suggest that the severity of word retrieval deficits in aphasia may be related to deficits in attentional shifting, though this does not imply directional cause between attentional shifting and anomia. This interpretation should be considered tentative, as the path analysis was exploratory and was conducted in a limited sample. Moreover, the association between attentional blink magnitude and word retrieval deficits emerged in the path analysis when T1 accuracy was included but this association was not observed in the initial linear regression. Therefore, these findings should be viewed as hypothesis-generating and require replication in larger samples before firm conclusions can be drawn about their stability and generalizability. If this relationship continues to be evident in future studies, it would provide support for an attentional theory of language deficits in aphasia, which proposes that language processing, including lexical retrieval, is achieved through attentional control processes and, therefore, one mechanism of impairment may involve attentional processes such as attention shifting (Faroqi-Shah and Gehman, 2021; Hula and McNeil, 2008). Future investigations are needed to further explore this relationship, as a greater understanding of it has the potential to provide new avenues for development and refinement of effective treatment approaches.

### 4.5 Limitations and future directions

This study was designed based on a prior investigation with individuals with brain lesions (Rizzo et al., 2001) and has yielded novel and interesting data that suggest that it is worth further exploring attentional shifting and its role in language function for people with aphasia. There are some aspects of this study, however, that limit its generalizability and provide direction for future research. First, the sample size is relatively small and limits the ability to fully analyze all of the cognitive-linguistic factors that may contribute to attentional shifting in PWA. Though the small sample size in this study is typical of aphasia research (Mohapatra and Dash, 2023), further research with larger samples is necessary to corroborate and expand these findings. In addition, this study was limited in that the cognitive-linguistic measures used in the pre-testing battery did not provide a large enough range of scores to justify statistical analysis between attentional blink magnitude, processing speed, and other measures such as short-term memory and working memory (see Appendix for exploratory analyses). Future studies should incorporate a broader and more sensitive battery of cognitive and language measures that would allow a more rigorous examination of the relationship between attentional shifting and other measures. Furthermore, we recognize that the present study did not examine language experience or multilingualism as explanatory factors for differences in performance on the experimental tasks or on the pre-testing battery, as these factors were not the focus of this study. This is a limitation, particularly because we did not collect detailed data on language use, language dominance, or degree of bilingual experience. Participants in the present sample varied in their language backgrounds, ranging from some exposure to languages other than English to full immersion in multilingual environments. Future studies should collect more detailed language history data to examine whether bilingualism or broader language experience contributes to linguistic and nonlinguistic attentional shifting in PWA.

Another limitation of this study concerns the timing parameters used to assess attentional blink effects in PWA. The intervals between T1 and T2 used in Task 1 spanned up to 1,200 ms. This range was the same as used by Rizzo et al. (2001) and ensured that the protocol remained feasible and was not overly fatiguing for participants. However, given that PWA demonstrate evidence of delayed response times (e.g., Evans and Quique, 2021; Faroqi-Shah and Gehman, 2021) and delayed lexical activation (e.g., Love et al., 2008), these intervals may not have fully captured the time course of attentional shifting in PWA; PWA may require additional time to consolidate T1 and successfully shift attention to T2. If this is the case, then further exploration of a broader range of T1-T2 intervals may provide additional useful information about the time needed for PWA to successfully shift their attention. Finally, given the apparent confound of visual complexity or linguistic coding in the nonlinguistic task used in this study, a nonlinguistic paradigm that alleviates those concerns is needed to understand how attention shifting impairments in aphasia are related to more domain-general cognitive processes in this population.

## 5 Conclusion

This study contributes to the literature demonstrating evidence of impaired attention shifting and processing speed in aphasia (e.g., Chiou and Kennedy, 2009; Helm-Estabrooks, 2002; Kuptsova et al., 2023; Peristeri et al., 2020) and expands upon this literature by using methods with high temporal sensitivity to index rapid attention shifting, which may be critical for rapid, fluent lexical access and overall language processing. While we did not find a direct relationship between attention shifting and lexical access, we found preliminary evidence for a potential association that suggests a path forward to understanding the mechanisms of word retrieval and their impairment in aphasia. Additionally, we identified factors that challenge the ability to measure attentional blink for nonlinguistic stimuli that can inform future research on the roles of language-specific and domain-general attention shifting skills in PWA. Continuing this line of research will move us toward a more comprehensive understanding of how to optimally address word retrieval and other language processing impairments in aphasia treatment.

## Data Availability

The datasets presented in this study can be found in online repositories. The names of the repository/repositories and accession number(s) can be found at: https://osf.io/kuncv/overview?view_only=03cc3a1ce3c947b5bab85a6a616a5d73.

## Appendix: Exploratory models

We explored linear regressions between cognitive-linguistic pre-tests, attentional blink magnitude, and processing speed. While most were not significant, there was a significant relationship between nonverbal working memory (as measured by the Pointing Span Task Backward) and the attentional blink magnitude, such that higher blink magnitudes were associated with longer nonverbal working memory spans [*β* = 0.35, *t*(10) = 4.71, *p* < 0.001].
